# Health status and access to health services of female prisoners in Greece: a cross-sectional survey

**DOI:** 10.1186/s12913-016-1506-3

**Published:** 2016-07-11

**Authors:** Mary Geitona, Stella-Olga Milioni

**Affiliations:** Department of Social and Educational Policy, University of Peloponnese, Damaskinou & Kolokotroni Str, 20100 Corinth, Greece

**Keywords:** Imprisonment, Female prisoners, Health status, Access to healthcare, Greece

## Abstract

**Background:**

Self-reported health status of prisoners’ population and access to health services during incarceration have not been adequately explored in Greece. The purpose of this study was to assess female prisoners’ health status and access to healthcare in the Attica detention center “Korydallos”.

**Methods:**

A cross-sectional survey was carried out in 2014. A semi-structured questionnaire was developed, including questions on the prisoners’ socio-demographic characteristics, self-reported health status before and after incarceration, access to and quality of the provided health services. Inmates who were in solitary confinement, suffered from serious psychiatric problems, or were unable to understand the Greek language were excluded from the study. Data were collected via personal interviews and information received was anonymized. Descriptive statistics and bivariate analyses were used. The difference between self-reported general health status prior to and during detention was analyzed by Wilcoxon test, while the relationship between health status and access to healthcare was explored with coefficient Spearman's rho. Statistical significance was set at p ≤ 0.05 level and statistical analysis was performed using the software SPSS 19.

**Results:**

Of the 135 prisoners, 101 participated in the study. 60.4 % mentioned a moderate or poor health status, while the respective percentage before detention was 32.7 %. Health status deterioration and poor mental health were reported by more than half of the respondents. Additionally, the vast majority expressed raised feelings such as sadness, anxiety or discomfort which affected negatively their everyday life while in prison. Regarding risk factors during imprisonment; tobacco consumption has increased by 16.6 % and 7.9 % of the sample admitted having used drugs. Moreover, the access to and the quality of provided health services in prison were described as poor/ very poor by 46.5 % and 49.5 %, respectively. A significant correlation between the access to and the quality of health care services and health status was observed.

**Conclusion:**

Healthcare provision at the “Korydallos” prison is not satisfying since the access to as well as the quality of healthcare are not adequate. Imprisonment leads to deterioration of self-reported health status. Our findings should constitute a starting point for further research in order to introduce more effective interventions aiming at meeting prisoners’ health needs.

## Background

Incarceration rates have increased by approximately 25-30 % over the last 15 years [1]. In 2013, more than 10.2 million people were incarcerated in rehabilitation centers as either pre-trial prisoners or serving sentence [1]. Female prison population has also increased by an average of 16 % worldwide in the years 2006–2012 [2].

Prisoners’ health represents one of the major challenges for public health especially since the increasing incarceration rates has a direct impact on prisoners’ overall health status. It has been reported that inmates experience high levels of physical and mental health problems relating to the prison environment, which is characterized by isolation, communal life, violence, insecurity, threat and overcrowding [3]. The high prevalence of prisoners’ health related problems seems to be attributed to numerous socio-economic characteristics, such as poverty, low income, and low education level [4–7]. Furthermore, empirical data reveal that prisoners make use of health services in prisons 3 to 4 times more frequently than the general population, mainly due to the high levels of morbidity associated with the worsening of their physical and mental health status, as well as the everyday life while in prison [8, 9].

Prisoners’ health status, access to health services and satisfaction associated with the quality of the provided healthcare are issues that have drawn researchers’ attention internationally. It has been widely reported that prisoners have generally poor access to health services and usually do not receive the appropriate care [10–12]. The high rates of chronic and mental disorders, as well as the high prevalence of sexually transmitted diseases during imprisonment, are significant public health issues that require measures aiming at improving prisoners’ access to health services [13–17].

Prisoners’ health status, as well as the incidence of different diseases varies by gender and age group [18–21]. In women, significant biological changes occur, which are more critical and evident in some age groups in relation to men [22, 23]. Women have more frequent and intense mental health problems compared not only to male prisoners but also to the general population [22, 23]. It should also be noted that mental illness is frequently present both as a cause and as a consequence of the imprisonment [23]. In addition, female prisoners have been found to be mostly dependent on alcohol or drugs [24], 10 times at greater risk of harming themselves than men [25] and three times more likely to have suffered physical or sexual abuse before their imprisonment in comparison with the general population [26–29]. The majority has experienced a head or a traumatic brain injury at some point in their life [30, 31].

In Greece, limited work has been conducted in the field of assessing prisoners’ health status and access to healthcare. The need for such a study was considered imperative given that incarceration impacts negatively their overall health status, their family environment as well as their everyday life after release [32]. The aim of this study was to explore issues around the health status and access to health care services provided to female prisoners incarcerated in the “Korydallos” facility.

## Methods

### Study design and sample selection

Given that women in Greek penal institutions reach approximately 5 % of the total prison population [2], there are only two female detention centers in the country, one in the prefecture of Attika (named “Korydallos”) and one in Central Greece (“Elaionas”). A cross-sectional survey was carried out in the female detention department of “Korydallos” from January to December 2014. The research was undertaken by the University of Peloponnese, which was granted an entry permit to the prison by the Ministry of Justice. Of a total of 135 incarcerated women in “Korydallos”, the female prisoners who were not in solitary confinement, who did not suffer from serious psychiatric problems and were able to understand the Greek language, were invited to participate in the study. The recruitment process was undertaken by prison’s employees who were social workers, specially trained for the purpose of the study by the university research team. The prisoners were informed on the study objectives and scope and were asked if they agreed to participate. The list of participants was subsequently given to the University research team and the interviews were scheduled.

### Study instrument

A questionnaire was developed based on a review of the international literature [33–35]. Due to lack of a standardized questionnaire for prisoners’ health status and access to health care assessment, a pilot research was carried out on a convenience sample of 12 female prisoners from the target population, in order to evaluate its clarity, comprehensiveness and validity. Pilot research was conducted in January 2014 and feedback was incorporated into the final questionnaire. The questionnaires were completed during face to face interviews with the inmates. The interviews were conducted by a specially trained and experienced researcher of the University of Peloponnese, with over 5-years experience in managing vulnerable population groups. Interviews were conducted in Greek without the prison’s personnel presence. All questionnaires were anonymized, in order to ensure confidentiality.

The questionnaire included three sections. The first section consisted of questions regarding the prisoners’ socio-demographic and detention characteristics, focusing on age, ethnicity, marital status, number of children, education, employment status at prison entry, income/ financial support, frequency of visits during incarceration, as well as reason, frequency and duration of imprisonment. Self-reported health was investigated in the second section, which included questions with regards to prisoners’ general, mental and dental health status. Respondents were asked about their general health status prior to entering and while in prison. Health risk behavior related questions, such as sexual behavior, lifestyle and addiction were also included in this section. The third section referred to the access and utilization of health services. More specifically, respondents were asked to answer questions on the availability, the ease of access to primary and secondary care, the frequency and the specialty of visits, as well as the adequate supply of outpatient and inpatient healthcare provision. This section also included questions on women’s satisfaction with the quality of the provided health services.

It should be noted that there are no female prisoners’ hospitals in Greece, thus women may receive hospital and outpatient care through the National Health System (NHS). In the “Korydallos” detention center, emergency and outpatient care is also provided by once-a-week visiting physicians, a dentist and four registered nurses employed on a full-time basis.

Self-reported health status before and after incarceration, access to and quality of the provided health services were explored with a 5-point Likert scale, where responses ranged from “very poor” to “very good”. Also, a 5-point ordered-category instrument ranging from “very little” to “very much” was used to measure the frequency of experiencing their feelings such as sadness, anxiety and the effect of them on prisoners’ daily life habits. Smoking history and current status, lifetime history of drugs use, prior to and during incarceration, experience in prostitution (paid sexual activity), sexual abuse and treatment for sexually transmitted diseases were also explored with the use of closed-ended questions.

### Statistical analysis

Statistical analysis was performed through descriptive statistics and bivariate analysis. The results are presented as absolute (n) and relative (%) frequencies for the nominal and ordinal variables and as mean values for the quantitative variables. The proportions of answers poor/ very poor or good/ very good were presented as a cumulative result. The variables are not normally distributed, so for the statistical differences and associations, non-parametric tests were performed. In particular, in order to investigate the difference between general health status before and during detention, the Wilcoxon test was used. The relationship between health status (dependent variables) and access to health services (independent variables) was explored via a correlation analysis with Spearman's rho given that the variables were ordinal. For final interpretation of statistical significance, *p* = 0.05 was used. The statistical analyses were conducted on S.P.S.S. 19 (Statistical Package for Social Sciences) [36].

## Results

Of a total of 135 female prisoners, 101 met the inclusion criteria and were enrolled in the study, reaching a 74.8 % response rate. More specifically, female prisoners who were in solitary confinement (*N* = 3), suffered from serious psychiatric problems (*N* = 14) or were unable to understand the Greek language (*N* = 17) were excluded from the study. The majority (76.2 %) was Greek, with a mean age of 37.5 years (Table 1). 52.5 % of the study population was unmarried and 61.4 % reported having children (of whom 38.6 % of children were below the age of 18 years and 25.7 % were living with one of their grandparents). 35.6 % and 31.7 % had compulsory or secondary education, respectively and 58.4 % was employed at the time of imprisonment.

**Table 1:** Sociodemographic characteristics of the sample

	N	%
Mean Age = 37.5 (S.D.11)		
Citizenship		
Greek	77	76.2
Other	24	23.8
Marital status		
Unmarried	53	52.5
Married	19	18.8
Divorced	21	20.8
Widow	8	7.9
Children		
Yes	62	61.4
No	39	38.6
Occupation before detention		
Employee	59	58.4
Student	2	2.0
Unemployed	26	25.7
Pensioner	3	3.0
Housework	9	8.9
Other	2	2.0
Education level		
Compulsory	36	35.6
Secondary	32	31.7
Tertiary	24	23.8
Postgraduate	9	8.9

85.9 % of the study population was financially supported by their family during incarceration, while the main reasons of imprisonment were drug trafficking, theft/robbery and financial reasons (Table 2). Financial imprisonment reasoning refers to debts to banking system, public sector and pension funds, to uncovered checks and to defalcation. It should also be noted that more than half (58.4 %) of the study population were pre-trial prisoners. For the overwhelming majority (83.2 %) of respondents, this was their first incarceration.

**Table 2:** Detention characteristics of the prisoners

	N	%
Detention reasons		
Financial reasons	26	25.7
Theft/Robbery	27	26.7
Drug trafficking	29	28.8
Injuries	2	2.0
Murder	7	6.9
Other	10	9.9
Imprisonment status		
Serving sentence	59	58.4
Pre-trial	42	41.6
Previous incarceration		
Yes	17	16.8
No	84	83.2
Financial support		
Family	85	85.9
Relatives	2	2.0
Friends	8	8.1
Others	4	4.0

The mean time in prison for women serving sentence was 1.8 years, while for pre-trial prisoners it was 8.5 months. 76.2 % of the respondents stated that their family members (mainly parents and children) paid regular visits during their detention; frequency of visits was once a week and more than once a week in 35.1 % and 28.4 % respectively.

### Health status

**Fig. 1:**
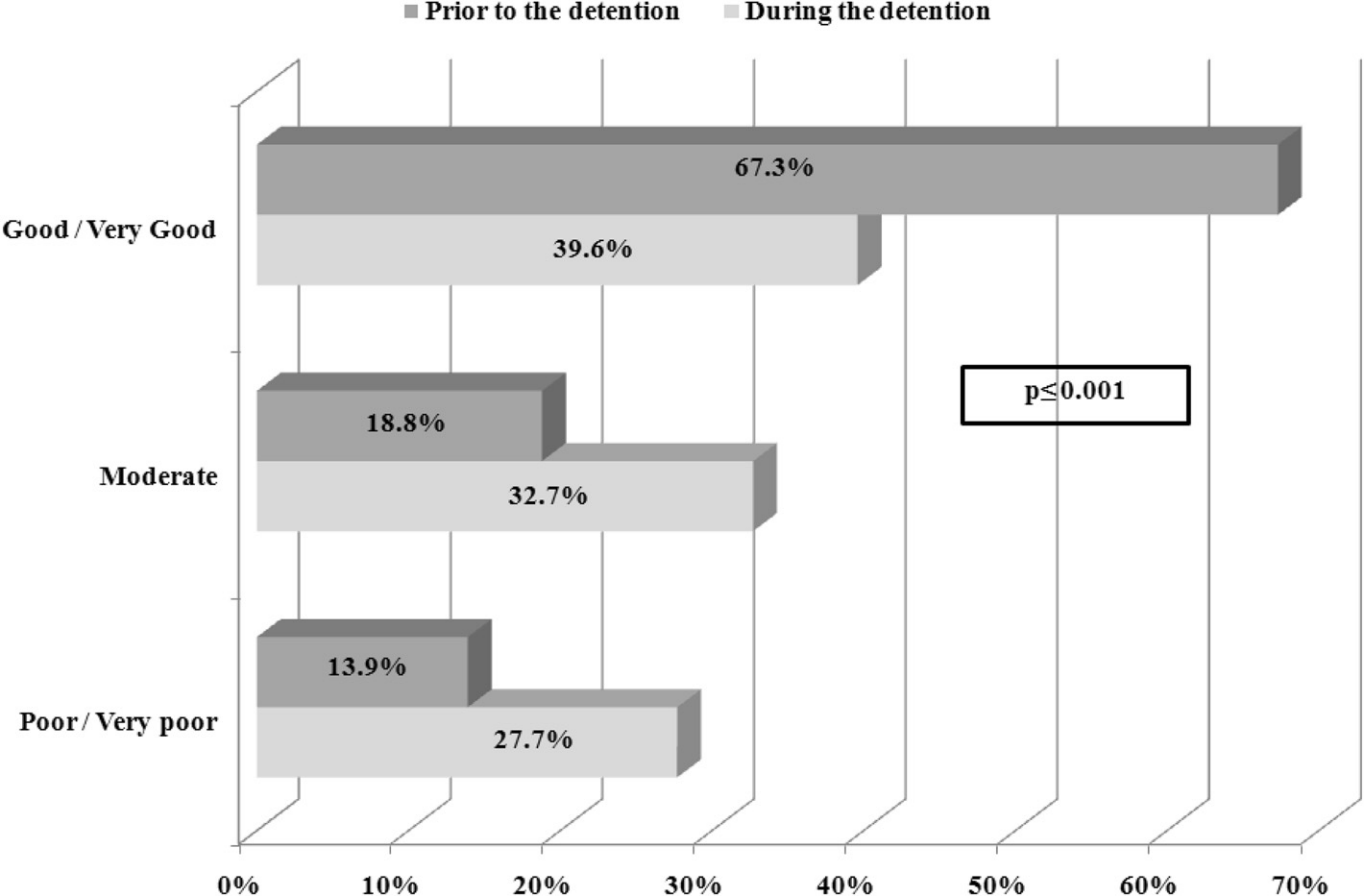
Self-reported general health status prior to and during the detention. This figure presents how prisoners’ rated their self-reported general health status prior to and during the detention

Female prisoners were asked about their overall health status, as well as their dental and mental health, before and during detention. The majority of the sample (67.3 %) rated their health status, prior to the detention, as good/ very good, while 67.4 % described their current health status as moderate or poor/very poor (Fig. 1) and 63.4 % reported that their health had worsened during detention. Also, 43.5 % of participants stated their current dental health as good/ very good, while 55 % stated their mental health as poor/ very poor (Table 3). The latter is further supported by the fact that 82.2 % reported that they have been feeling “much/ very much” sadness, anxiety or discomfort during the detention. 86 % also mentioned that these feelings affected negatively their everyday life.

**Table 3:** Self-reported mental health status during the detention

	Mental health	Dental health
Good /very good	24.0 %	43.5 %
Moderate	21.0 %	27.7 %
Poor/very poor	55.0 %	28.8 %

### High risk behavior

Female prisoners were asked about the tobacco, alcohol and drug use prior to and during their detention. 70 % of the respondents were smokers with the majority (67.2 %) smoking more than 21 cigarettes daily before detention, whereas this percentage increased to 83.8 % during detention. 66.7 % stated that they did not consume alcohol before detention. Moreover, 31.7 % of the study population replied that they had made use of drugs before their detention while 7.9 % admitted having used drugs during imprisonment. However, 3 % of women were unwilling to answer. Also, 6.9 % replied positively as to having experience in prostitution (paid sexual activity), as well as having received medical treatment for a sexually transmitted disease. As far as sexual abuse is concerned, 14 % had been sexually abused before detention.

### Access to and utilization of health services

**Fig. 2:**
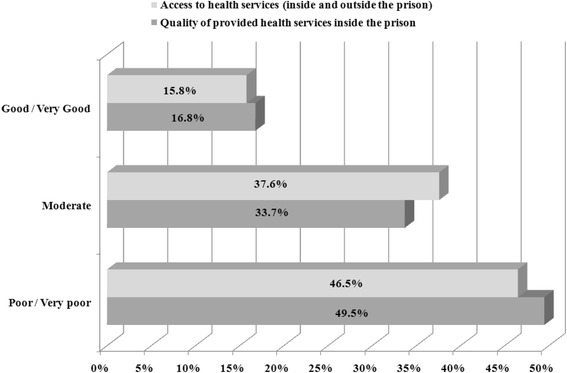
Access to and Quality of health services. This figure presents how prisoners’ described the access to health services both inside and outside the prison, as well as the quality of provided health care services in prison

Female prisoners were asked to assess their access to healthcare as well as the quality of the health services provided during their imprisonment. The medical specialties that prisoners have been consulting most frequently inside the prison were general practitioners (75.2 %) and psychiatrists/psychologists (52.5 %), while 46.5 % reported that they receive medication relating to their mental health problem. As far as diagnostic tests are considered, only 36.3 % of the study population had been tested for HIV, and 42.6 % and 43.6 % for Hepatitis B and C, respectively. Regarding health services utilization outside the prison, 36.6 % stated that they had been visiting the NHS hospitals’ outpatient units, 18.8 % urban health centers while the same percentage had been hospitalized. The access to health services both inside and outside the prison, as well as the quality of provided health care services in prison were described as poor/very poor by 46.5 % and 49.5 % of the respondents, respectively (Fig. 2).

### Correlation analysis

Statistically significant differences were found between general/dental/mental health and access to healthcare (both prior to and during detention), as well as the health services provided while in prison (Table 4). The increase in access to and quality of health care services was linearly positively correlated with the improvement of health status.

**Table 4:** Correlation analysis between health status and access to, quality of health services

		Self-reported general health status	Self-reported dental health status	Self-reported mental health status
Access to health services (inside and outside the prison)	Correlation Coefficient	0.373^a^	0.245^b^	0.339^a^
	Sig. (2-tailed)	0.001	0.013	0.001
Quality of health services in prison	Correlation Coefficient	0.329^a^	0.266^a^	0.318^a^
	Sig. (2-tailed)	0.001	0.007	0.001

^a^Correlation is significant at the 0.01 level, ^b^ Correlation is significant at the 0.05 level

## Discussion

To the best of our knowledge the present study is the first attempt to assess prisoners’ self-reported health status before and during incarceration and their access to health services in Greece. According to the study results, approximately 3 out of 10 female prisoners rated their general health status as poor compared to that before incarceration, while two-thirds reported that their health status, and particularly their mental health, worsened during imprisonment. Half of the participants reported that access to health care services did not meet their needs and the quality of provided healthcare in prison was poor.

Most of our findings are consistent with the international literature. Prisoners’ poor health status compared to that before detention, as well as a high prevalence of mental disorders and mental health deprivation, have been reported in various studies [5, 10, 37–47]. More specifically, the rate of serious mental illnesses among prisoners has been reported to be 3 to 5 times higher than that of the general population [42]. Another study in Italy showed that prisoners with a mean age of 40 years rated their health as poor [40]. Furthermore, our study findings regarding Greek female prisoners’ views and opinions on the access to and quality of health services also seem to be consistent with other studies reporting that inmates are provided a low level of inpatient care during incarceration, since they do not have a direct equivalent access to healthcare in the community and they cannot, as the general population, choose their health service providers [8, 9, 37, 48–50].

Our study also showed that a small percentage of prisoners had either experience in prostitution or had been sexually abused before detention. The literature has shown higher percentages with respect to domestic violence (50 %) and sexual abuse (33 %) [27–29]. In the European Union, women in prison are more likely to suffer from sexually transmitted diseases, due to their prior either high-risk behavior, such as prostitution, or traumatic experiences, such as rape or sexual abuse [37].

Despite the fact that diagnostic tests are compulsory for prisoners according to the Greek Penal Code [51], only 40 % of the respondents had been examined for sexually transmitted diseases during detention. Similar findings have also been reported in other surveys [10, 37, 52], highlighting the increased risk of spreading these diseases to other prisoners and to the community after release.

There are some potential limitations in this study. The first limitation refers to the lack of a standardized questionnaire; nevertheless, the tool developed for data collection was based on international literature review. Moreover, a limitation may have been introduced by the use of a self-reported instrument for assessing health status, access to and quality of health care services [40, 53]; however our results seem not to be underestimated, because these questions were arranged on similar level of responses and also high correlations were found. In addition, there is the possibility of a recall and selection bias since the mean age of our sample was young, that might influence prisoner’s self-assessed health status. However, Greek female prisoners are mostly young [54], thus this limitation should be considered as minimal and does not threaten robustness of results. It should be noted that despite the fact that our findings come from one out of two female prisons in Greece, they are not generalizable to the country given that the second female prison is more crowded (approximately 370 prisoners).

Overall, the level of health of female prisoners has been found to be moderate to poor and deteriorating during incarceration. Women in prison suffer from mental health disorders and have limited access to health care services. These findings should be taken into consideration from policy and decision makers in order to improve the quality of health care services provided to this patient population, which constitutes a very sensitive part of the Greek society.

## Conclusions

The findings suggest that imprisonment is associated with deterioration of self-reported health status and limitations to access to health care services. The study calls the attention of decision makers with regards to the provision and quality of healthcare. Our findings should constitute a starting point for further research in order to prevent the effects of incarceration on prisoners’ health and to introduce more effective interventions aiming at meeting their health needs.

## Abbreviations

Not applicable
